# Gender Perception From Gait: A Comparison Between Biological, Biomimetic and Non-biomimetic Learning Paradigms

**DOI:** 10.3389/fnhum.2020.00320

**Published:** 2020-08-27

**Authors:** Viswadeep Sarangi, Adar Pelah, William Edward Hahn, Elan Barenholtz

**Affiliations:** ^1^Department of Electronic Engineering, University of York, York, United Kingdom; ^2^Center for Complex Systems and Brain Sciences, Florida Atlantic University, Boca Raton, FL, United States

**Keywords:** motion perception, biological motion, gait, machine learning, human perception, machine perception

## Abstract

This paper explores in parallel the underlying mechanisms in human perception of biological motion and the best approaches for automatic classification of gait. The experiments tested three different learning paradigms, namely, biological, biomimetic, and non-biomimetic models for gender identification from human gait. Psychophysical experiments with twenty-one observers were conducted along with computational experiments without applying any gender specific modifications to the models or the stimuli. Results demonstrate the utilization of a generic memory based learning system in humans for gait perception, thus reducing ambiguity between two opposing learning systems proposed for biological motion perception. Results also support the biomimetic nature of memory based artificial neural networks (ANN) in their ability to emulate biological neural networks, as opposed to non-biomimetic models. In addition, the comparison between biological and computational learning approaches establishes a memory based biomimetic model as the best candidate for a generic artificial gait classifier (83% accuracy, *p* < 0.001), compared to human observers (66%, *p* < 0.005) or non-biomimetic models (83%, *p* < 0.001) while adhering to human-like sensitivity to gender identification, promising potential for application of the model in any given non-gender based gait perception objective with superhuman performance.

## Introduction

A person’s gait carries information about the individual along multiple dimensions. In addition to indicating biologically intrinsic properties, like gender and identity, the gait of a person changes dynamically based on their emotional state (Pollick et al., 2002) and state of health (Cesari et al., 2005). Humans are adept at identifying whether a given sparse motion pattern is biological or not (Johansson, 1973, 1976) as well as detecting properties such as gender or mood. However, the origin of these abilities remains unclear. One the one hand, the ability to distinguish biological from non-biological motion appears at a very young age (Fox and McDaniel, 1982), suggesting there may be some expert-system capacities present at birth. Indeed, some theorists have suggested that biological motion perception served as an evolutionary and developmental precursors to the theory of the mind (Frith, 1999). However, recognition of biological motion could also be attributed to generic learning systems that are trained with experience (Fox and McDaniel, 1982; Bertenthal and Pinto, 1994; Thompson and Parasuraman, 2012), where adults have been shown to be able identify biological motion which was synthetically created using machines (while the infants could not), indicating a learning system that tunes itself based on experience. One way to address this is to compare the behavior of human observers to computational learning models of different types that can be trained “from scratch,” i.e., without specialized mechanisms pre-tuned to the properties of biological motion. This provides the opportunity to assess whether generic learning models trained on biological motion stimuli serve as a reasonable model of human behavior or whether additional mechanisms, such as pre-tuned expert systems, should be posited. In addition, we classify the computational models into two groups: (1) biomimetic models, that functionally replicate the neural learning systems in humans, especially biological memory, and (2) non-biomimetic models, which utilize statistical techniques to identify discerning features in data for classification. For clarification, we use the term “biomimetic” as, the study of the structure and function of living things as models for the creation of materials or products by reverse engineering (Farber, 2010). A strong resemblance between humans and biomimetic models, but not with non-biomimetic models, would provide further evidence that the mechanisms underlying human biological motion perception are well captured by a generic learning model. To do so, we compared performance in a simple binary biological motion classification task (gender recognition) and compared human performance to a range of computational learning models.

### Biological Models

Johansson (1973, 1976) first demonstrated that human observers were sensitive to biological motion through point light displays representing joints of a human walkers. Despite their sparsity, human observers readily interpreted the stimuli as human gait. Subsequent research with point-light walkers demonstrated the ability of humans in the identification of familiar people (Loula et al., 2005). In case of unfamiliarity, observers could extract certain general categories such as approximate age and gender (Kozlowski and Cutting, 1977; Barclay et al., 1978; George and Murdoch, 1994; Lee and Grimson, 2002; Pollick et al., 2005) with significantly higher than chance accuracy. In case of gender identification, humans achieved the best performance when presented with the stimuli in the coronal plane, due to the prevalence of dynamic cues (George and Murdoch, 1994). However, the biological nature of human perception hinders its replicability and transferability. The learning is highly variable, volatile and susceptible to fatigue, illness and mortality, leading to the need for automation of gait classification. Automation of gait classification has been extensively studied with a high focus on performance outcome through classification accuracy. However, mimicking human perception closely would ensure versatility of the artificial classifier, enabling the same classifier to be used in other non-gender related gait classification tasks.

### Machine Learning Models

Machine learning (ML) models can be trained to identify the relevant attributes in gait such as gender with high speed and fidelity. The models can broadly be divided into two categories: (1) Memory based models comprised of artificial neural networks (ANN) such as the Long Short Term Memory (LSTM) cells (Graves and Schmidhuber, 2005), which operate on time series data, and (2) Static models, such as the Random Decision Forests (RDFs) (Kaur and Bawa, 2017) and Support Vector Machines (SVMs) (Huang et al., 2014), which operate on static data. The LSTM model shall be referred to as the “biomimetic” models crediting the functional implementation of the biological neural network and memory using artificial neurons, while the SVM and RDF shall be referred to as the “non-biomimetic” models. Prior studied have promising results in terms of the ability of biomimetic ML in being able to mimic human observation of gait (Pelah et al., 2019; Sarangi et al., 2019; Stone et al., 2019). However, an in-depth exploration of the above mentioned models and direct comparison to human observers on the same stimuli has not been conducted.

#### Biomimetic Models

Artificial neural networks aim to mimic the flow of information in the biological brain by creating a network of neurons, based on the perceptron model (Rosenblatt, 1958). Recurrent neural networks (RNN), an implementation of the ANN, simulates the memory capabilities of the human brain, by creating an additional feedback loop for processing latent network state along with new data (Mikolov and Zweig, 2012). RNNs operate on a sequence of vectors as input data. The sequence resembles the time series information and the vector represents the features of the input at each timestamp. However, RNNs suffer from vanishing and exploding gradient rendering them ineffective in processing long sequences (Graves and Schmidhuber, 2005). LSTM cells overcome this problem by introducing additional gates in the network to regulate the flow of information, enabling them to remember relevant temporal patterns over long periods of time (Graves and Schmidhuber, 2005).

#### Non-biomimetic Models

The non-biomimetic ML techniques considered in this paper learn to classify information based on (1) linear separability, following non-linear projections, and (2) reduction of information entropy based on feature thresholds. SVM models learn to fit a linear hyperplane to maximize the separation between the classes in the training dataset. Decision trees, learn to classify information based on the learned numerical thresholds of features. RDF is a collection of randomly initialized decision trees with majority vote of the cohort considered as the predicted class. SVMs and RDFs accept static representations of data as input and thus cannot process temporal sequences of information, unlike humans and LSTMs.

## Data Collection

Forty one consenting healthy adults (26 male, 15 female) between the ages of 18 and 50 years old were recorded walking on the treadmill. Participants volunteered and received credit toward a participation grade for their class. Appropriate consent forms were signed and anonymity maintained. Gait data was recorded as spatiotemporal three-dimensional joint trajectories for 20 tracked joints of the body. The tracked points on the walker’s skeleton included the head, neck, shoulders, elbows, wrists, fingertips, mid spine, back, hips, knees, hips, ankles, and toes. The collection of the joint positions formed a static frame. Data was captured at 24 frames per second, each frame represented by 60 numbers (3D coordinates of 20 joints) and a corresponding timestamp of capture of the frame. Data was recorded for 6 sessions per participant. Each session consisted of a minute of walking on the treadmill at a self-selected speed followed by a minute’s rest. The joints were extracted utilizing a consumer-level time-of-flight based RGB-D sensor, the Microsoft Kinect v2. The sensor provides an anthropomorphic representation of the human skeleton through 3D joint coordinates. The sensor was placed approximately 1.5 m in front of the treadmill with the front board removed to avoid issues with occlusion. The ML based skeletal motion capture method mentioned in Shi et al. (2018) is used for capturing the PLD representation of the biological motion of the walkers. When compared with the state-of-art optical motion tracking methods [such as Vicon (Clark et al., 2012; Pfister et al., 2014)], the anatomical landmarks from the Kinect-generated point clouds can be measured with high test-retest reliability, and the differences in the interclass coefficient correlation between Kinect and Vicon are <0.16 (Clark et al., 2012, 2013; Pfister et al., 2014; van Diest et al., 2014). Both systems have been shown to effectively capture >90% variance in full-body segment movements during exergaming (van Diest et al., 2014). The validity of biological motion captured using the Kinect v2 sensor is established in Shi et al. (2018) with human observers through reflexive attentional orientation and extraction of emotional information from the upright and inverted PLD.

## Experiment 1: Biological Models

Studies have shown humans to require no longer than two complete gait cycles to correctly identify gender from human gait (Huang et al., 2014). In terms of duration, this translates to less than 2.7 s of walking animation. Although humans can decipher biological motion from point light animation of walking human figure within 200 msec, at least 1.6 s of stimulus is required for significantly above chance performance. This experiment aims to establish the change in gender identification performance in humans as a function of increasing duration of stimulus exposure.

### Method

#### Biological Model

Fifteen female and six male healthy observers with age ranging from 20 to 43 years old, participated in the experiment. All had some experience of biological motion displays, although none had been required to make judgments about gender.

#### Stimuli

**FIGURE 1:**
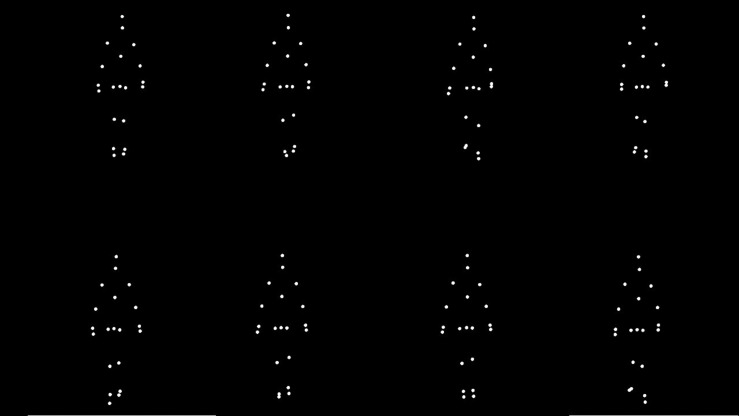
Point light representation of a walking stimulus at eight different stages of a gait cycle.

A PC-compatible computer monitor with a high performance raster graphics system displayed stimuli on an Iiyama ProLite B2283HS color monitor (1920 × 1080 resolution, 60 Hz refresh rate). Human figures were defined by 20 circular white dots of 5 pixel radius overlaid on a black background, located on the head, neck, shoulders, elbows, wrists, fingertips, back, spine, hips, knees, ankles, and toes. None of the dots were occluded by other subjective parts of the figure. Animated sequences were created by placing the dots at the three-dimensional trajectory of each of the 20 tracked joints, and temporally sampling the coordinates to produce 24 static frames per second, as shown in Figure 1. The stimulus size was 6 degrees wide and 8 degrees long for the whole frame, including zero (black) padding. A degree of visual angle is defined as the subtended angle at the nodal point of the eye. The actual walking clip was 2.5 degrees wide and 4 degrees long. The presented stimuli was height normalized to fit the given aspect ratio, to prevent the observers from identifying gender based on height of the animated walker.

When the static frames were played in quick succession, a vivid impression of a walking person emerged. There was no progressive component to the walking animation, thus the human figure appeared to walk on an unseen treadmill with the walking direction oriented toward the observer. The height range for males was 144–208 cm, and for females was 129–152 cm. None were notably over- or underweight (see Table 1). The x and z component were sampled to display the walker in the coronal plane to emphasize lateral sway and maximize the provision of dynamic cues to the observer (Barclay et al., 1978; Troje, 2002). The recorded gait sequences were converted into an animation sequence in the same fashion to be presented as visual stimuli. The observers were seated in a well-lit room in front of the monitor and had access to a standard computer mouse for interaction. The randomly chosen walker stimuli were presented for exposure durations of 0.4, 1.5, 2.5, and 3.8 s, followed by an on-screen prompt in the form of two buttons requesting the observer prediction of binary gender through a mouse click on either of the labeled buttons. Following the response from the observer, the next stimulus was presented. A total of 200 walking clips were shown per observer per exposure duration and the responses recorded for each.

**TABLE 1:** Description of the walking subjects taking part in the stimulus set.

	Height (cm)	Weight (kg)	Age (years)
Male	176.23 ± 32.43	80.49 ± 2.86	26.06 ± 6.42
Female	128.56 ± 23.51	73.3 ± 4.59	21.29 ± 1.23

### Results

Human observers correctly identified 63% of all the trials across all exposure durations, *t*(20) = 7.8, *p* < 0.001, two tailed. All *t*-tests reported in this paper are two-tailed, unless otherwise indicated. Chance performance for the binary gender classification is 50% as expected. Correct identification at 0.4 s, which consisted of a quarter of a step cycle, was above chance at 60%, *t*(20) = 3.7, *p* < 0.01, conforming with (Barclay et al., 1978; Troje, 2002), however, was in disagreement with (Barclay et al., 1978). This could be attributed to the presentation of the stimulus in the coronal plane as opposed to the sagittal plane (Sarangi et al., 2019), leading to higher emphasis on the dynamic cues. Performance at 1.5 s is 66%, *t*(20) = 3.8, *p* < 0.005, which is higher than the performance at 2.5 s of 61%, *t*(20) = 4.8, *p* < 0.001. Barclay et al. (1978) explains this anomalous phenomenon due to an additional partial step at 2.5 s by highlighting the preferred perception of velocity over positional cues, where sensitivity to gender identification decreases mid-swing in the gait cycle. Humans were able to identify gender with highest accuracy at 3.8 s with 69%, *t*(20) = 3.4, *p* < 0.01. Details of the results obtained have been listed in Table 2. Overall, the performance of the human observers taking part in the experiment conforms to the results of perceptual experiments in literature (Barclay et al., 1978; Troje, 2002), providing a reliable baseline for comparison with the biomimetic perception on the same stimulus set.

**TABLE 2:** Gender identification accuracy in % as a function of exposure duration of the stimulus.

Model/Stimulus Duration	0.4 s	1.5 s	2.5 s	3.8 s
Biological (Human)	60 (*p* < 0.01)	66 (*p* < 0.005)	61 (*p* < 0.001)	65 (*p* < 0.05)

In summary, human observers were able to identify gender from gait with significantly above chance performance from moving dots presentations of joints, while conforming with existing human perception literature. There is a significant increase in gender identification performance between 0.4 and 3.8 s of stimulus exposure duration. The increased gender sensitivity at 1.5 s is attributed to the prevalence of dynamic, velocity based cues at the phase of the step cycle corresponding to that time (George and Murdoch, 1994), thus demonstrating the preference of humans toward dynamic velocity based cues compared to structural position based cues for gender identification.

## Experiment 2: Biomimetic Models

Long short term memory network’s capability to process a temporal sequence of data aims to mimic the temporal pattern recognition capabilities of humans. The learning gates inherent in the network parallel the short and long term memory of the human brain, enabling the network to remember the relevant temporal pattern while ignoring patterns that don’t contribute toward the classification objective. This experiment aims to present an LSTM network with the temporal evolution of joint trajectories during human gait and train it for gender identification to evaluate for resemblance with human observers.

### Method

#### Biomimetic Model

**FIGURE 2:**
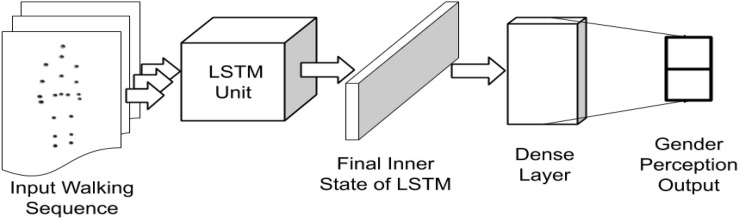
Implementation of the LSTM network architecture for processing gait sequences.

A standard LSTM model consisting of 128 hidden states in the cell (as shown in Figure 2), is initialized. The cell state weights were initialized as a random normal distribution. The final cell state was ReLU activated (Maas et al., 2013) and connected to an affine output layer, which represented the one-hot labeled gender identity of the walker during training. During testing, the output layer represented the prediction values. The error of prediction was evaluated using a cross-entropy function (de Brébisson and Vincent, 2015) for updating of the weights using an Adam optimizer (Kingma and Ba, 2014) based on the error differentials and a learning rate of 0.001. The most probable output was taken as the class label during prediction. 10 LSTM models, mimicking 10 random human perceptions, were generated for inferring the gender from gait input.

### Data Input

The three-dimensional trajectories of each of the 20 tracked joints were concatenated to form a vector representation of a static frame with a cardinality of 60, representing the location of the head, neck, shoulders, elbows, wrists, fingertips, mid-back, hips, knees, ankles, and toes. Gait input to the model consisted of a sequence of vector representations of subsequent static frames, sampled at 24 frames per second. Joint trajectories were size normalized (Troje, 2002) and standardized with a zero mean and unit standard deviation. Model training sessions included, initialization of the model weights, prediction of the output probabilities based on the gait input, propagation of the prediction error and updating the network weights. Model training was executed in batches of 50 and repeated for 100 epochs. Input sequence durations mirrored the exposure durations in the corresponding human perception experiment and varied incrementally for 10 durations from 0.4 to 3.8 s in steps of 0.4 s (10 static frames). 10-fold cross validation was carried out to ensure model generalizability and a total of 250 gender predictions were obtained per input sequence duration. The models trained per session per duration are stored locally for future analyses.

### Results

**FIGURE 3:**
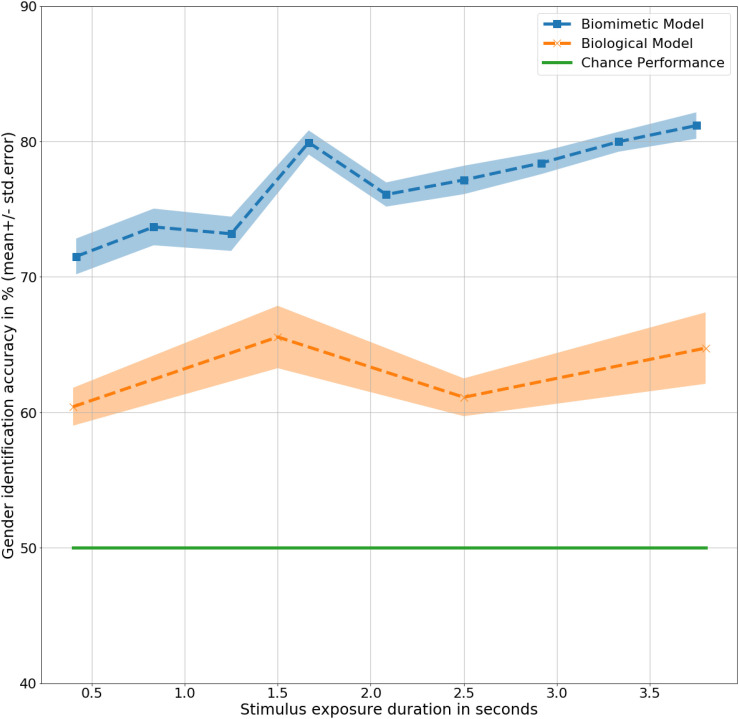
Gender identification performance in mean ± standard error % by the models as a function of exposure duration in seconds.

Long Short Term Memory models correctly identified 76% of all the gait inputs presented across all the input durations, *t*(9) = 9.2, *p* < 0.001. Chance performance remains same at 50%. Correct identification at a quarter of a step cycle at 0.4 s was 71%, *t*(9) = 5, *p* < 0.001, higher than the same with human observers, *F*(9,20) = 3.6, *p* < 0.1. The difference in performance indicates a higher inference capacity from a limited amount of available data. The inference performance increases slightly with increase in the amount of information available from 0.4 to 3.8 s, *F*(9,9) = 2, *p* < 0.1. At 3.8 s, the model correctly identified gender with 81% accuracy, *t*(9) = 9.6, *p* < 0.001, considerably higher than human observers, *F*(9,20) = 9, *p* < 0.01. Generalizing across all the input (or exposure) durations, the biomimetic model identified gender with a significantly higher accuracy than the human observers, *F*(9,20) = 39.9, *p* < 0.001. Details of results obtained for the LSTM model have been presented in Table 3 with the corresponding trend plotted in Figure 3. As shown in the figure, mean performance peaks temporarily at 1.6 s (halfway completion of one gait step) with 79% accuracy, *t*(9) = 10.1, *p* < 0.001 suggesting a dependence on dynamic and velocity cues similar to humans at 1.5 s.

**TABLE 3:** Gender identification accuracy as a function of exposure duration of the stimulus with *p* < 0.001 for all the durations.

Model/Stimulus Duration	0.4 s	1.5 s	2.5 s	3.8 s
Biological (Human)	60 (*p* < 0.01)	66 (*p* < 0.005)	61 (*p* < 0.001)	65 (*p* < 0.05)
Biomimetic (LSTM)	71 (*p* < 0.001)	73 (*p* < 0.001)	77 (*p* < 0.001)	81 (*p* < 0.001)

In summary, the biomimetic LSTM model performed significantly better than chance in gender classification from 3D moving point representations of human gait. There was a significant increase in gender identification accuracy from 0.4 to 3.8 s of gait information exposure, corresponding to humans. The increased gender sensitivity at 1.6 s could be attributed to an inherent sensitivity to dynamic velocity based cues in LSTM networks for gender identification, similar to humans. One could argue that the presentation of the skeleton stimulus as facing toward the camera could potentially limit real-world applications. However, although specific deployments would need to be assessed, the availability of 3D data could be leveraged to apply a simple preprocessing rotational step to the skeleton to correct for any misalignment in global skeletal configuration.

## Experiment 3: Non-Biomimetic Models

The non-biomimetic models, unlike humans and LSTMs are capable of analyzing static data only. Their reliance on the principles of linear separability and information entropy to create rules for classification, resembles expert systems. The models require a static representation of the spatiotemporal gait data for gait classification. Thus data was represented as, (1) static descriptions of the temporal signals, and (2) extracted metrics used in a clinical setting to describe gait for diagnosis and rehabilitation monitoring. In this experiment, we evaluate the SVMs and RDFs on the two static representations of gait data for resemblance with human observation.

### Method

#### Non-biomimetic Models

Support vector machines are designed to linearly separate a set vectors to achieve maximum classification accuracy, whereas DTs learn to classify by choosing optimal split conditions of attributes to minimize ambiguity in classification. SVMs with linear (SVM-Linear), radial basis function (SVM-RBF) with gamma as 0.99 and sigmoid (SVM-Sigmoid) kernels were evaluated. The non-biomimetic models also included RDFs of 10 randomly generated ID3 DTs (Kaur and Bawa, 2017) with a minimum requirement of two samples for splitting and a maximum of three features for splitting consideration.

### Data Input

Gait data, originally represented as a temporal sequence of vectors, was described with four first-order statistics, namely, minimum, maximum, mean, and standard deviation of each dimension in the multi-dimensional time series signal. The temporal sequence duration of the signal was varied from 0.4 to 3.8 s in steps of 0.4 s (10 frames). The resulting static dataset was normalized and standardized to have a mean of zero and unit standard deviation.

The study was conducted in conjunction with the Cambridge University Hospitals, thus the gait metrics utilized for clinical gait analysis in the gait analysis laboratory were mirrored as static representations of gait. 12 spatiotemporal metrics, including, stride length, cadence, single-double support, stance-swing phase ratio, speed of walking and knee flexion for each leg during stance and swing were provided as input feature sets to the static learning models. All the features were standardized to have a mean value of zero and a standard deviation of one. The features were further normalized to lie within the [−1, 1] range for uniformity and to discourage the models from learning the gender from the structural information and to rely solely on the gait dynamics.

### Results

**FIGURE 4:**
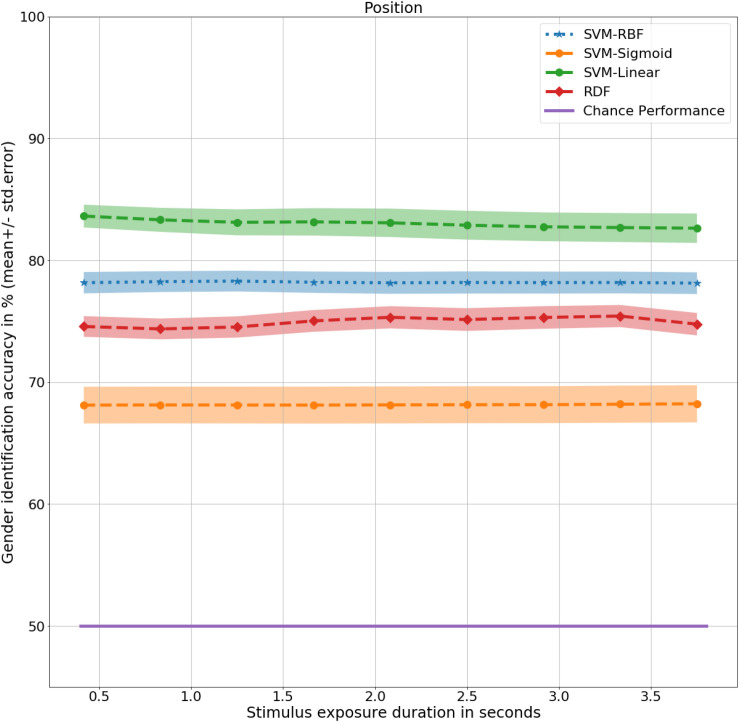
Gender identification performance of non-biomimetic models as a function of duration of gait data used to generate the static representation. The shaded region around the central mean line represents the standard error in performance.

At 0.4 s, the RDFs and SVMs of all the kernels were able to identify gender with significantly better than chance performance with RDF at 75%, *t*(9) = 9, *p* < 0.001, SVM-Linear at 84%, *t*(9) = 12, *p* < 0.001, SVM-RBF at 78%, *t*(9) = 10, *p* < 0.001, and SVM-Sigmoid at 68%, *t*(9) = 4, *p* < 0.01, as shown in Figure 4. There was no significant difference in performance in the non-biomimetic models between 0.4 and 3.8 s of duration, unlike humans and LSTM models. Gender sensitivity remained similar across all the durations of gait input, demonstrating a dynamic cue agnostic learning mechanism. Performance output has been detailed in Table 4. All the non-biomimetic models performed close to chance performance. The best performing out of the cohort was the SVM with radial basis function with a gender identification accuracy of 59%, *t*(9) = 1.8, 0.1 < *p* < 0.2, followed by the RDF classifier with an accuracy of 59%, *t*(9) = 1.5, 0.1 < *p* < 0.2. The statistical significance of the results is yet to be established with the collection of more data. However, the motivation for testing the non-biomimetic models further is reduced based on the results.

**TABLE 4:** Performance of non-biomimetic models in % correctly identified gender.

Model/Duration	0.4 s	1.5 s	2.5 s	3.8 s
SVM-Linear	83.8 (*p* < 0.001)	83.5 (*p* < 0.001)	82.8 (*p* < 0.001)	82.5 (*p* < 0.001)
SVM-RBF	78 (*p* < 0.001)	78 (*p* < 0.001)	78.5 (*p* < 0.001)	77.9 (*p* < 0.001)
SVM-Sigmoid	68 (*p* < 0.01)	68.1 (*p* < 0.01)	68.2 (*p* < 0.01)	68.2 (*p* < 0.01)
RDF	74.9 (*p* < 0.001)	74.6 (*p* < 0.001)	74.2 (*p* < 0.001)	73.5 (*p* < 0.001)

In summary, the biomimetic models that were trained on the four static representations of the temporal signals performed significantly better than chance and the corresponding models trained on clinical gait metrics performed at or below chance performance. Notably, there was no significant change in performance with increasing duration of exposure of gait input. In addition, there was no change in sensitivity to gender identification with increasing availability of information at different phases of the step cycle. Both the above characteristics have been observed in humans and LSTMs, suggesting a deviation of the non-biomimetic models from a common learning mechanism shared between humans and LSTMs. Humans also possess the trait of being sensitive to dynamic velocity based cues for gender identification. In order to explore the existence of the trait in the artificial classifiers, the next experiment trains models on velocity cues exclusively, to evaluate the change in performance.

## Experiment 4: Biomimetic Models With Velocity Cues

Humans are known to rely on dynamic velocity based cues when determining gender from gait. This experiment focuses on training the biomimetic and non-biomimetic models on velocity cues exclusively. An increase in performance would determine a common trait shared with the humans.

### Method

#### Biomimetic Model

The LSTM model architecture remains the same as mentioned in Experiment 2. The weights initialization, training regime and data input style is maintained. The only difference is brought about because of the difference in the data input being provided.

#### Biomimetic Data Input

In Experiment 2, gait was represented as a temporal evolution of the positional joint trajectories. For this experiment, temporal derivatives of the gait of the walkers were used for generating corresponding velocities of the joints. The positional data was smoothed with a 5-frame moving average filter before calculating the derivatives for adjacent frames. The training was performed as mentioned in Experiment 2 and 10-fold cross validation ensured generalizability of the results.

#### Non-biomimetic Model

The non-biomimetic models namely the SVM-Linear, SVM-RBF, SVM-Sigmoid and the RDF remain the same as the previous experiment, however, the training data provided is a static representation of the temporal derivative of the data provided in Experiment 3.

#### Non-biomimetic Data Input

The positional joint trajectories of 20 tracked joints are smooth using a 5-frame moving average filter followed by a temporal derivative of the smoothed signal to obtain the three-dimensional velocity of the joint trajectories. The result is represented as four static attributes, namely, minimum, maximum, mean and standard deviation of the temporal signal. The temporal duration of the signal is varied from 0.4 to 3.8 s in steps of 0.4 s (10 frames). The static representation is normalized between [−1, 1] and standardized to have zero mean and unit standard deviation. The resulting dataset is used for training and testing of the non-biomimetic models with a 10-fold cross validation of the walkers.

### Result

#### Biomimetic Model

**FIGURE 5:**
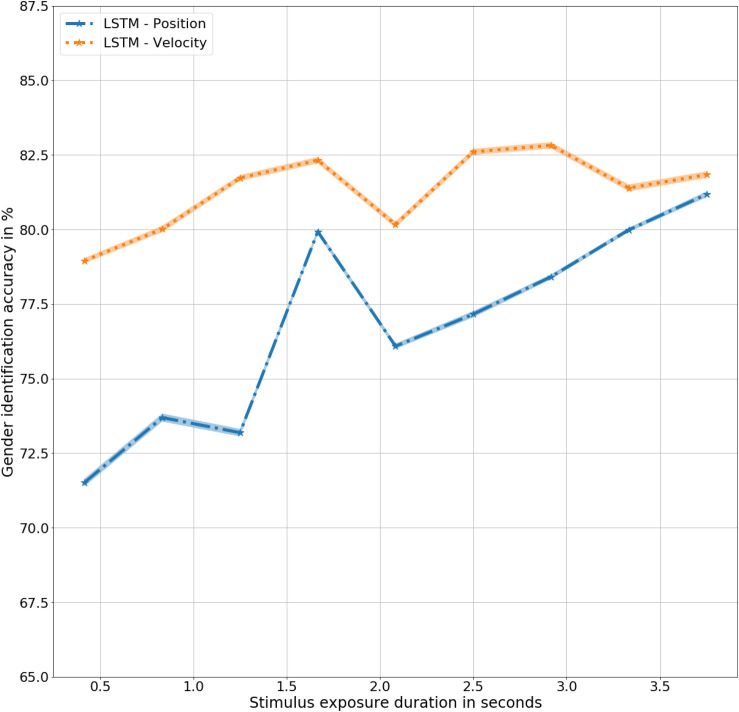
Gender identification performance in mean ± standard error % by LSTM models trained with Position and Velocity as a function of exposure duration in seconds.

The biomimetic LSTM model trained with three-dimensional velocity (LSTM – Velocity) achieved an overall accuracy of 81%, *t*(9) = 9.4, *p* < 0.001, significantly better than the human observers, *F*(9,20) = 82, *p* < 0.001 for all durations as well as the LSTM model trained with three-dimensional positions (LSTM – Position) of joint trajectories, *F*(9,9) = 5.6, *p* < 0.05. The model achieves its highest accuracy at 2.8 s of exposure with an accuracy of 83%, *t*(9) = 9.4, *p* < 0.001. As shown in Figure 5, the lack of velocity based cues is noticed at 2.2 s in LSTM – Velocity, corresponding to the similar lack dynamic cues at the same time in LSTM – Position, demonstrating the dependence of the LSTM network on velocity while determining gender.

#### Non-biomimetic Models

**FIGURE 6:**
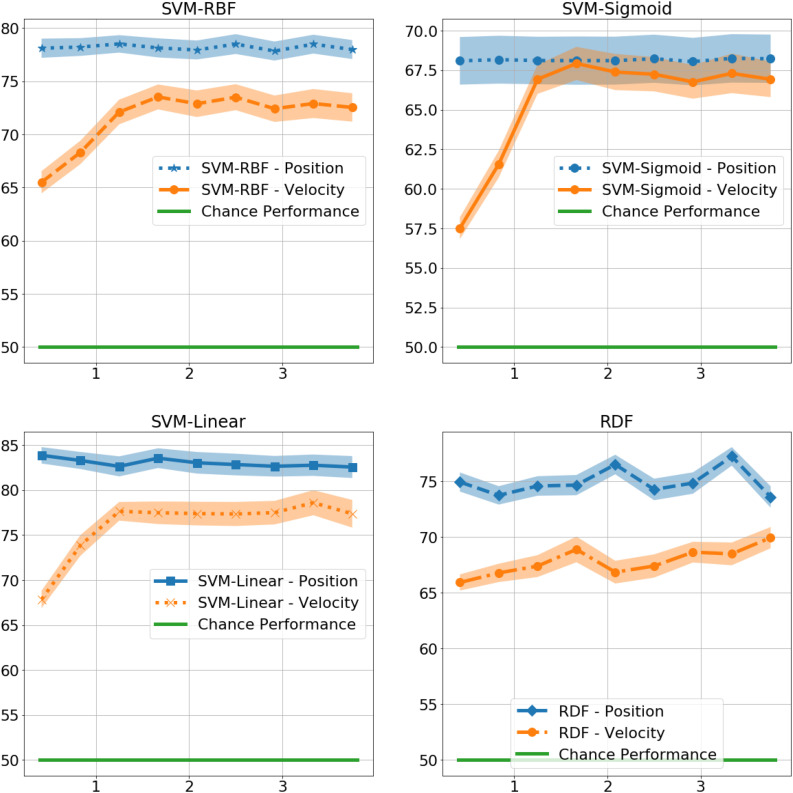
Gender identification performance of non-biomimetic models trained with Position and Velocity information, as a function of duration of gait considered for extracting the static representation.

As shown in Figure 6, the performance of the non-biomimetic models decreased significantly upon training with velocity data, compared with the position data (details provided in Table 5). SVM-Sigmoid demonstrates a significant increase in performance with increasing duration of gait data provided, *F*(9,9) = 4.5, *p* < 0.05 with the best accuracy of 67%. However, the change in performance is not statistically significant in other SVM and RDF models. The behavior goes against the expected biological behavior and the results demonstrated by the LSTMs, denoting a loss of performance in a form of data which is biologically more conducive to gender identification.

**TABLE 5:** Gender identification performance of non-biomimetic models trained with Position and Velocity gait information and the difference in performance between the corresponding models denoted through *F*-test.

Model/Gait Data	Position	Velocity	*F*-Test, *F*(9,9)
SVM – Linear	83%	76%	37 (*p* < 0.001)
SVM – RBF	78%	72%	52 (*p* < 0.001)
SVM – Sigmoid	68%	65%	5 (*p* < 0.05)
RDF	75%	68%	149 (*p* < 0.001)

As shown in Figure 6, the performance of the non-biomimetic models decreased significantly upon training with velocity data, compared with the position data (details provided in Table 5). SVM-Sigmoid demonstrates a significant increase in performance with increasing duration of gait data provided, *F*(9,9) = 4.5, *p* < 0.05 with the best accuracy of 67%. However, the change in performance is not statistically significant in other SVM and RDF models. The behavior goes against the expected biological behavior and the results demonstrated by the LSTMs, denoting a loss of performance in a form of data which is biologically more conducive to gender identification.

In summary, training biomimetic and non-biomimetic models with joint velocities produced contradicting results when compared to the corresponding models trained with joint positional trajectories. The result of the non-biomimetic models goes against the established human dependence on dynamic velocity based cues for gender identification. The results obtained through the biomimetic LSTM networks not only conforms to the expected biological behavior, but also demonstrates the shared gender sensitivity trend observed at different phases of the walk cycle between the biological and biomimetic models. However, one could argue that the provision of three-dimensional gait information to the biomimetic models but the two-dimensional screen based input to the humans could cause a gap in direct comparison between the models, making it difficult to draw parallels between the learning mechanisms. In the next experiment, the biomimetic LSTM model is trained and tested on two-dimensional gait information by omitting the depth information, which the humans had to infer from the screen.

## Experiment 5: Biomimetic Models With Two-Dimensional Input

Long Short Term Memory networks have demonstrated a close resemblance with the human observers, making it conducive to draw parallels between the learning mechanisms. However, humans observed the moving point animations on a two-dimensional screen for gender identification while the LSTMs were provided with three-dimensional motion information. This experiment trains the LSTMs with two-dimensional gait information to form a direct comparison with the human observers, without assuming any depth inference capabilities of humans.

### Method

#### Biomimetic Model

The LSTM model architecture is similar to the previous experiment. However, the number of inputs are reduced by a third, owing to the loss of the z-components of the joint trajectories. The weights initialization, training regime and data input style is maintained. The only difference is brought about because of the difference in the data input being provided.

#### Data Input

In Experiment 2, gait was represented as a temporal evolution of the positional joint trajectories. The three-dimensional trajectories of each of the 20 tracked joints were concatenated to form a vector representation of a static frame with a cardinality of 60. This experiment maintains the same data style but omitting the z-component, modifying the vector representation to have a cardinality of 40. The gait information is varied from 0.4 to 3.8 s in steps of 0.4 s (10 frames). The resulting data is normalized between [−1, 1] and standardized to have zero mean and unit standard deviation.

### Result

**FIGURE 7:**
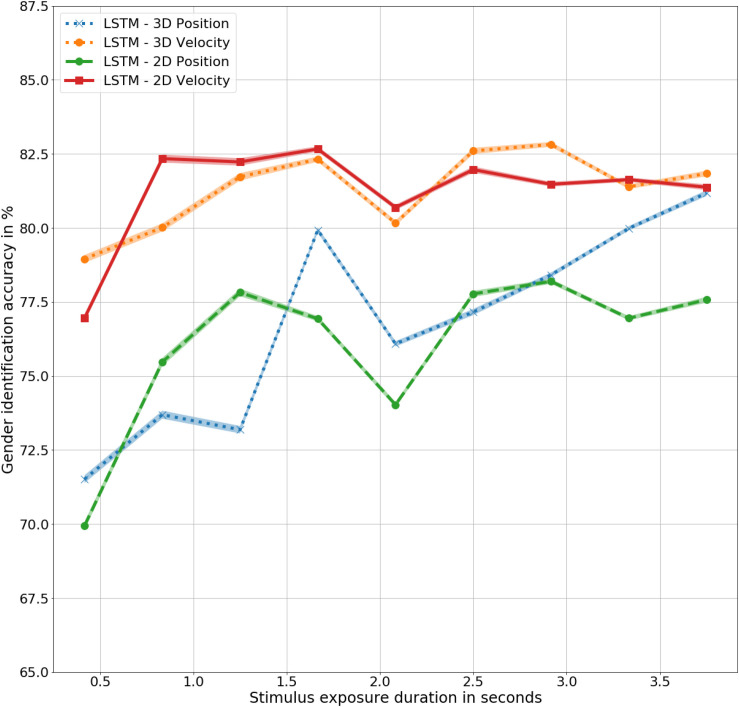
Gender identification accuracy using biomimetic LSTM models trained in three- and two- dimensional position and velocity representations of the joint trajectories.

There is no statistically significant difference in the outcomes of the LSTMs trained with three- and two-dimensional joint trajectories, in corresponding position and velocity information, as shown in Figure 7. However, the difference between the models trained with corresponding 3D and 2D values is significant with *F*(9,9) = 6, *p* < 0.05 for LSTM – 2D Position and LSTM – 2D Velocity, with accuracies of 76% and 80%, respectively. Notably, the performance of the models trained with 3D and 2D velocities are significantly higher than the models trained with the corresponding position representations. The two-dimensional models also demonstrate the unique gender sensitivity trait possessed by human observers and the biomimetic models trained with three-dimensional representations of gait, further supporting the close resemblance of the LSTM models with humans.

In summary, the loss of depth information didn’t cause any significant change in performance accuracy all the traits that were shared with humans and corresponding biomimetic models trained with three-dimensional gait information were maintained, further supporting the close resemblance of the artificial model with human perception.

## Discussion

Biomimetic models share the following characteristics with human observers, which the non-biomimetic models either do not share or have the opposite trait of: (1) Increase in gender identification performance with increasing temporal availability of gait information, (2) Preference toward dynamic velocity based cues as opposed to structural position based cues for gender identification, leading to higher performance in the former data type, and (3) Unique trend of gender sensitivity during different phases of the walk cycle. The results support a closer congruence in biological motion perception between humans and biomimetic models, compared to non-biomimetic models. Additionally, the close resemblance confirms the ability of the biomimetic learning models in emulating human learning dynamics. This study presents a correlation between the human visual perception of motion as well as the biomimetic memory-based neural network. This, however, does not necessarily indicate causation, but provides a bidirectional mode of understanding human perception using models as simple as the LSTM. The objective of finding correlations between human performance and models for more general use, in a non-arbitrary manner, while minimizing model parameters and assumptions is accomplished. It is interesting that a simple LSTM model is able to capture certain dynamics of human perception of gender from gait from an input-output perspective. This is consistent with previous findings for the inversion effect, a failure to accurately classify gender from gait in upside down stimuli (Sarangi et al., 2020). Observing such correlations between human and biomimetic models encourages further investigations that may help to elucidate both types of “black box” models.

From an application standpoint, the results encourage the potential of using biomimetic models for gait classification. Although the paper uses gender identification as the gait classification objective, the resemblance with human observers may widen the scope of application to non-gender related classification tasks as well. The results could also be further improved through a more conservative approach to cross-validation such as the leave-one-out cross validation. Interestingly, the availability of 3D skeletal data tests the effect of rotation of the skeleton and its correlation with gender identifiability for human observers. The same should not affect the performance of the biomimetic model as a simple preprocessing rotation step could correct for any misalignment in global skeletal configuration. Finally, although treadmill walking may not be congruent with over ground walking, the experiment establishes the ability of the machine based models to extract relevant features from spatiotemporal skeletal data.

The non-biomimetic models on the other hand, require explicitly hand crafted features which may not be applicable to a generic classification task. As shown in Experiment 3, the clinical gait metrics representation of gait didn’t possess information about gender, while the static four attribute representation possessed enough information about gender for a better than chance performance. The transferability of the same features toward a new objective (such as person identification) is brought into question and requires further experiments.

## Conclusion

Humans are highly adept at classification of gait for a multitude of objectives, from gender identification to clinical diagnosis, while relying on a common learning mechanism of spatiotemporal perception of gait. This paper applies a number of ML models, each with a different mode of learning, to the classification of gender from gait, comparing their performance to that of human observers under controlled conditions. The analysis aims to identify, firstly, specific correlations between humans (biological), biomimetic and non-biomimetic learning models, and secondly, to find an artificial model that best resembles human performance to potentially generalize its application to other gait classification tasks. Results are analyzed in terms of performance profiles and preferences of motion cues over structural cues. While not necessarily informative on underlying neural substrates in humans the findings demonstrate the parallel usefulness of the biomimetic approach. Although non-biomimetic and biomimetic models exhibit comparable levels of performance, the biomimetic models are more generalizable in not requiring hand-engineering of features for a given application. Biomimetic are also useful for modeling human perception at least from an input-output perspective, while, conversely, perceptual findings can improve the pragmatic effectiveness of models, contrasting with the synthetic approach that is typically employed in ML research.

## Data Availability Statement

The datasets generated for this study will not be made publicly available as there are privacy laws regarding data protection.

## Ethics Statement

The studies involving human participants were reviewed and approved by the Internal Review Board, Florida Atlantic University. The patients/participants provided their written informed consent to participate in this study.

## Author Contributions

VS and AP conceived of the presented idea. VS performed the computational experiments. WH performed the psychophysical experiments with supervision from EB. AP and EB helped fortify the argument in the manuscript. VS wrote the manuscript with help from EB and AP. All authors discussed the results and contributed to the final manuscript.

## Conflict of Interest

The authors declare that the research was conducted in the absence of any commercial or financial relationships that could be construed as a potential conflict of interest.
